# Alteration of colonic epithelial cell differentiation in mice deficient for glucosaminyl *N*-deacetylase/*N*-sulfotransferase 4

**DOI:** 10.18632/oncotarget.12915

**Published:** 2016-10-26

**Authors:** Tzu-Ming Jao, Ya-Lin Li, Shu-Wha Lin, Sheng-Tai Tzeng, I-Shing Yu, Sou-Jhy Yen, Ming-Hong Tsai, Ya-Chien Yang

**Affiliations:** ^1^ Department of Clinical Laboratory Sciences and Medical Biotechnology, National Taiwan University College of Medicine, Taipei, Taiwan; ^2^ Department of Laboratory Medicine, National Taiwan University Hospital, Taipei, Taiwan; ^3^ Laboratory Animal Center, National Taiwan University College of Medicine, Taipei, Taiwan; ^4^ Department of Surgery, Cardinal Tien Hospital, New Taipei City, Taiwan; ^5^ School of Medicine, Fu-Jen Catholic University, New Taipei City, Taiwan

**Keywords:** NDST4, tumor suppressor gene, heparan sulfate proteoglycan, Ndst4 knockout mouse

## Abstract

Glucosaminyl *N*-deacetylase/*N*-sulfotransferases (NDSTs) are the first enzymes that mediate the initiation of heparan sulfate sulfation. We previously identified NDST4 as a putative tumor suppressor in human colorectal cancer. In the study, we generated an *Ndst4* knockout (*Ndst4^−/−^*) mouse strain and explored its phenotypic characteristics, particularly in the development of colonic epithelial homeostasis. The Ndst4-deficient mice were viable and fertile, and their life spans were similar to those of wild-type littermates. No gross behavioral or morphological differences were observed between the *Ndst4^−/−^* and wild-type mice, and no significant changes were determined in the hematological or serum biochemical parameters of the *Ndst4^−/−^* mice. *Ndst4* RNA transcripts were expressed in the brain, lung, gastrointestinal tract, pancreas, and ovary. However, Ndst4-null mice exhibited no gross or histological abnormalities in the studied organs, except for the colon. Although no alterations were observed in the crypt length or number of proliferating cells, the *Ndst4^−/−^* mice exhibited an increased number of goblet cells and a decreased number of colonocytes in the proximal colon compared with the wild-type mice. Moreover, Ndst4 deficiency increased the basal level of apoptosis in the colonic epithelium. Taken together, we established, for the first time, an *Ndst4^−/−^* mouse strain and revealed the involvement of Ndst4 in the development and homeostasis of colonic epithelium. Accordingly, NDST4 in human colon might direct the biosynthesis of specific heparan sulfate proteoglycans that are essential for the maintenance of colonic epithelial homeostasis. Thus, the loss of its function may result in the tumorigenesis and progression of colorectal cancer.

## INTRODUCTION

Glucosaminyl *N*-deacetylase/*N*-sulfotransferases (NDSTs) are the first modification enzymes during the biosynthesis of heparan sulfate [1, 2], which is one of the sulfated glycosaminoglycans covalently attached to core proteins to form heparan sulfate proteoglycans (HSPGs). Syndecans and glypicans are widely expressed cell-surface HSPGs, whereas agrin, perlecan, and collagen XVIII are ubiquitously distributed in the extracellular matrix [3]. Because of the negative charges of sulfated heparan sulfate, HSPGs function as receptors and coreceptors that bind growth factors, cytokines, and chemokines [4, 5] and can thus maintain physiological homeostasis and moderate biological processes including development, morphogenesis, and inflammation [6–9]. Notably, the content and distribution of HSPGs are altered during tumorigenesis, and they have been implicated in the positive or negative aspects of cancer progression [10].

NDSTs are bifunctional enzymes that define the overall design of the sulfation pattern in HSPGs, which determines the ability of the heparan sulfate chain to bind target molecules. The ratios of *N*-deacetylase to *N*-sulfotransferase activity differ considerably among the four isozymes, causing them to exhibit varying substrate specificities [11]. Except for *Ndst4*, mice strains with targeted mutations in the other three *Ndst* genes have been established. Ndst1 deficiency results in neonatal lethality because of pulmonary hypoplasia and respiratory distress [12, 13]. Mice lacking Ndst2 function develop and reproduce normally with no detectable alterations in heparan sulfate biosynthesis. However, the connective tissue-type mast cells in these mice lack normally sulfated heparin and display defective storage of secretory granules [14, 15]. *Ndst3* knockout mice also develop normally but exhibit anxiety-related behavior and small alterations in high-density lipoprotein and total cholesterol. Nevertheless, Ndst3 deficiency does not lead to dramatic changes in the heparan sulfate composition in tissues [16].

The differentiation, homeostasis, and apoptosis of the colonic epithelium are modulated by complex processes, and the dysregulation of each process might increase the likelihood of developing colorectal cancer; most colorectal tumors arise sporadically through a combination of discrete mutations and chromosomal aberrations [17–19]. In a previous study, by using the loss of heterozygosity approach, we identified, for the first time, *NDST4* as a novel putative tumor suppressor gene associated with human cancer, and the loss of its function might be involved in the progression of colorectal cancer [20]. However, the physiological roles of NDST4 remain largely unknown.

To explore the functions of NDST4, particularly those related to the development and homeostasis of the colonic epithelium, in the present study, we first generated a *Ndst4* knockout (*Ndst4^−/−^*) mouse strain and then evaluated its physiological variations through analyses of viability, fertility, life span, morphology, and behavior, as well as through blood assays. In addition, we determined the histological alterations in 13 organs and the skeletal muscle of the *Ndst4^−/−^* mice through comparison with their wild-type (WT) littermates. We found that Ndst4 deficiency significantly increased the number of goblet cells and decreased the number of colonocytes in the proximal colon. Furthermore, the *Ndst4^−/−^* mice exhibited an increased basal level of apoptosis in the colonic epithelium. Thus, the findings of this study provide insights into the physiological role of NDST4 in the development and homeostasis of the human colonic epithelium, and suggest that NDST4 downregulation promotes the tumorigenesis and progression of colorectal cancer.

## RESULTS

### Ndst4 is moderately expressed in the gastrointestinal tract

**Figure 1:**
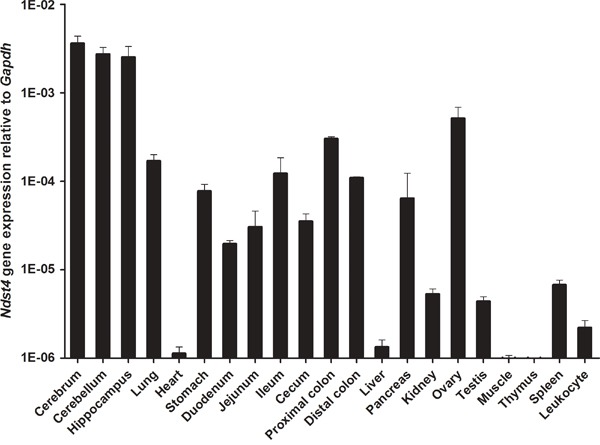
*Ndst4* gene expression in mouse tissue spectrum Eleven-week-old wild-type C57BL/6 mice were used to harvest organs and tissues for determination of *Ndst4* RNA transcripts by quantitative RT-PCR (n = 3). *Gapdh* gene was used as an internal RNA control. Data represent the mean ± SEM.

Although studies have revealed *Ndst4* expression in the mouse tissue spectrum by using reverse transcription-polymerase chain reaction (RT-PCR) [11, 16], its expression profile in the gastrointestinal tract has not yet been reported. In the present study, we determined, for the first time, that *Ndst4* RNA transcripts are expressed in the gastrointestinal tract (the stomach, duodenum, jejunum, ileum, and cecum, as well as the proximal and distal colon) harvested from 11-week-old C57BL/6 mice; we then compared the expression in the gastrointestinal tract with that in other organs and tissues by using quantitative RT-PCR. The results revealed that *Ndst4* was abundantly expressed in the cerebrum, cerebellum, and hippocampus and moderately expressed in the lung, gastrointestinal tract, pancreas, and ovary (Figure 1). However, *Ndst4* expression was negligible in the heart, liver, kidney, testis, skeletal muscle, thymus, spleen, and leukocytes.

### Generation of *Ndst4*-null mouse strain

**Figure 2:**
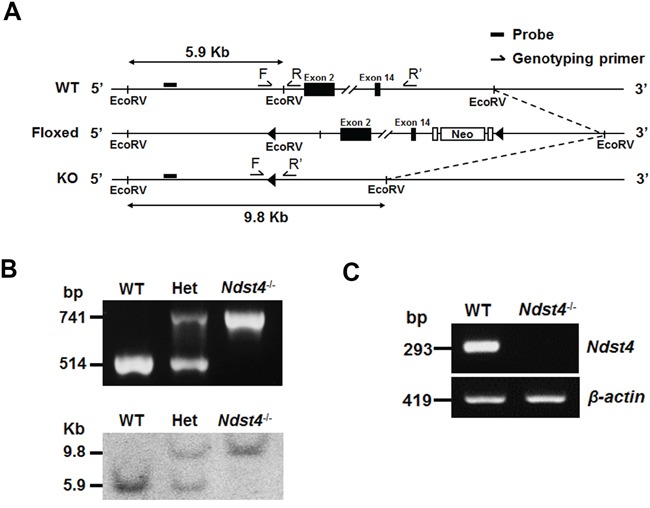
Disruption of the *Ndst4* gene by targeted homologous recombination **A.** Schematic representation of the *Ndst4* gene knockout strategy, including a map of the wild-type (WT), *LoxP*-floxed (Floxed) and expected targeted (KO) alleles. Exons 2 and 14 are shown as filled boxes. *LoxP* sites and *Frt*-flanked neomycin resistance gene (Neo) are indicated by triangles and open boxes, respectively. The probe and *Eco*RV sites are designed for Southern blot analysis. Primers of multiplex PCR, F, R and R’, are used for genotyping. **B.** Identification of the genotypes of the offspring from heterozygous intercrosses by using multiplex PCR (Upper panel) and Southern blotting (Lower panel). Het represents the heterozygous mutant mouse. **C.** Detection of *Ndst4* gene expression by using RT-PCR. The *Ndst4* mRNA was detectable in total RNA from the cerebrum of the WT but not *Ndst4^−/−^* mice. *β-actin* was used as an internal RNA control.

To elucidate the physiological roles of NDST4, we established a knockout mouse model of *Ndst4* by using the recombineering-based method [21]. Two targeting vectors were generated for sequential insertion of *LoxP* sites into the regions upstream of exon 2 and downstream of exon 14 through homologous recombination (Supplementary Figure S1). The deletion design eliminated the *N*-deacetylase and *N*-sulfotransferase activities of Ndst4. Briefly, the first vector targeting exon 2 (named Ndst4-1.1) was linearized and transfected into 129/Sv-derived embryonic stem (ES) cells through electroporation. The correct targeted clones were confirmed using Southern blotting, and the neomycin resistance gene was removed using Cre expression. Subsequently, the resulting ES cells were transfected with the second vector targeting exon 14 (named Ndst4-1.2), followed by Southern blotting confirmation. After Cre-mediated gene deletion of *Ndst4*, the selected ES cells were injected into C57BL/6 blastocysts to establish chimeric mice. Heterozygous mutant mice for the null mutation were generated by crossing founder male chimeras with C57BL/6 female mice. The genotypes of the weanlings obtained from heterozygous intercrosses were determined using multiplex PCR and were confirmed using Southern blotting of DNA extracted from tail biopsy samples (Figure 2A and 2B). Moreover, by using a primer pair targeting exons 2 and 4, we could not detect *Ndst4* RNA transcripts through RT-PCR of the cerebral RNA from the *Ndst4^−/−^* mice. However, the expected PCR products were obtained using the cerebral RNA from the WT mice (Figure 2C). The result confirmed that *Ndst4* mRNA was disrupted in the *Ndst4^−/−^* mice.

### Ndst4-deficient mice exhibit normal biological characteristics

To assess development and reproductive performance, 11 male and 22 female heterozygous (*Ndst4^+/−^*) mice were raised for intercrosses, and two male and four female *Ndst4^−/−^* mice were raised for incrosses. Genotyping of offspring derived from the heterozygous intercrosses revealed the expected Mendelian ratios of allelic distribution (25.6%, WT; 52.4%, *Ndst4^+/−^*; and 22.0%, *Ndst4^−/−^* mice; Supplementary Table S1). In addition, the normal mean litter sizes of 6.0 and 5.5 and the sex ratios of 0.85 and 0.94 were observed in the *Ndst4^+/−^* intercrosses and *Ndst4^−/−^* incrosses, respectively, indicating that both the male and female Ndst4-null mice developed normally and were fertile (Supplementary Table S1). Moreover, no differences were observed in life span between the *Ndst4^−/−^* mice and their WT littermates during the observation period, which is longer than one year for the youngest mice.

For phenotypic characterization, the modified SHIRPA protocol was implemented on a group of 27 *Ndst4^−/−^* mice and an age-matched group of 29 WT littermates (Supplementary Table S2). At the age of 8 weeks, Ndst4-null mice exhibited body weights similar to those of their control littermates (mean ± SD, 20.4 ± 2.8 g and 20.7 ± 3.4 g, respectively). Moreover, in another 57 subtests evaluating the morphology, behavior, sensory response, and athletic ability of the mice, the *Ndst4^−/−^* mice performed similarly to the WT controls, as indicated by the nonsignificant differences between the groups. This finding indicated that their morphology, muscle, motor neuron, spinocerebellar, sensory, and autonomic functions, as well as their neurological reflexes were within the normal range.

To evaluate the health status of the *Ndst4^−/−^* mice, we performed a hematological analysis and determined the metabolite and enzyme levels, lipid and renal function profiles, and electrolyte levels in the sera of 26 *Ndst4^−/−^* and 27 WT mice at 8–10 weeks of age. No significant differences were observed in the leukocyte count or differentiation between these two groups (Supplementary Table S3). The erythrocyte count (and related parameters) and platelet count were similar between the *Ndst4^−/−^* mice and their WT littermates. The results of the hematological analysis indicated that hematopoiesis and immune cell lineage differentiation occurred normally in the Ndst4-null mice. In addition, no significant differences were observed in the levels of serum biochemical parameters between the *Ndst4^−/−^* and WT mice, indicating that the *Ndst4* knockout mice exhibited normal body metabolism and normal liver and kidney functions (Supplementary Table S4).

### Ndst4 deficiency alters the cell lineage differentiation of the colonic epithelium

Morphological analysis for Ndst4-null mice at 10–11 weeks of age included macroscopic examination of body cavities, organs, and tissues and a histopathological analysis. Examination of all external and internal organs of 10 *Ndst4^−/−^* mice revealed no visible abnormalities compared with 10 age-matched WT littermates. For the histological examination, the sections prepared from 13 organs and skeletal muscle were stained with hematoxylin and eosin (H&E). The results showed that, except for the colon, no differences were observed between the *Ndst4^−/−^* and WT mice (data not shown).

**Figure 3:**
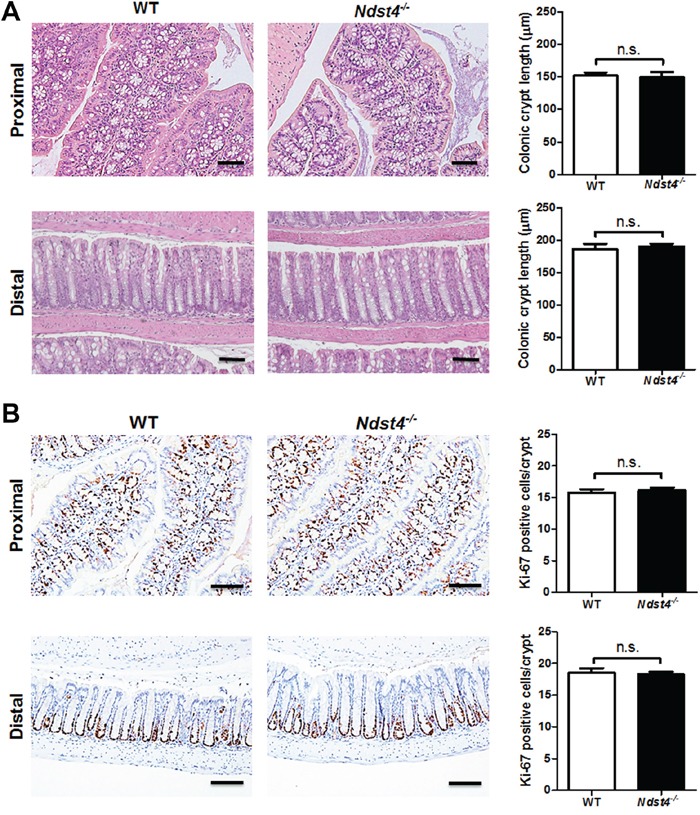
Ndst4 deficiency results in histological changes in the proximal colon Histological analysis was performed on the Swiss rolls made from the proximal and distal colonic segments of the *Ndst4^−/−^* mice and their wild-type (WT) littermates at 10–11 weeks of age (n = 10 for each group). **A.** Hematoxylin and eosin stained sections exhibit a notable increase of mucus-secreting goblet cells in the proximal colonic mucosa of the Ndst4-null mice compared with the WT mice. Quantification of crypt length shows no significant differences between the two groups. **B.** Proliferative activity in the colonic crypts was assessed using Ki-67 immunohistochemistry. Ki67-positive cells were located within the basal and surrounding cell layers of the crypts in both of the WT and *Ndst4^−/−^* mice. Fifty crypts were randomly selected in each mouse to measure crypt length and count ki-67-positive cells. Data represent the mean ± SEM of 10 mice per group. Scale bar, 50 μm. n.s., not significant.

**Figure 4:**
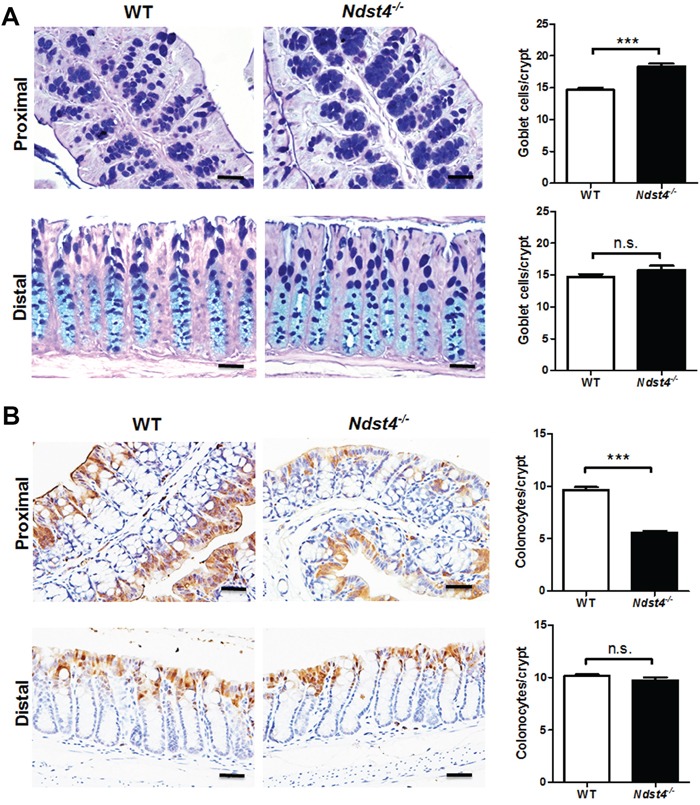
Ndst4 deficiency increases goblet cell differentiation along with a decrease of colonocytes in the proximal colon Histological analysis was performed on the Swiss rolls made from the proximal and distal colonic segments of the *Ndst4^−/−^* mice and their wild-type (WT) littermates at 10–11 weeks of age (n = 10 for each group). **A.** Goblet cells in the colonic mucosa were determined using Alcian blue/periodic acid-Schiff staining. In the proximal colon, the goblet cells staining blue were significantly increased in the Ndst4-null mice compared with the WT mice. **B.** Colonocytes in the colonic mucosa were determined using immunohistochemistry for carbonic anhydrase I. In the proximal colon, the colonocytes staining brown were significantly decreased in the Ndst4-null mice compared with the WT mice. Fifty crypts were randomly selected in each mouse to count goblet cells and colonocytes. Data represent the mean ± SEM of 10 mice per group. Scale bar, 20 μm. ***P < 0.001. n.s., not significant.

Regarding the colonic epithelium, the crypt length was similar between the *Ndst4^−/−^* mice and their WT littermates (Figure 3A). Moreover, Ki-67 immunohistochemistry revealed no significant differences in the number of proliferating cells between the *Ndst4^−/−^* and WT mice (Figure 3B). Notably, mucus bubbles, a characteristic of goblet cells, were increased in the proximal colon of the *Ndst4^−/−^* mice compared with their WT littermates (Figure 3A). The results suggested that Ndst4 deficiency correlates with cell differentiation but not proliferation of the colonic epithelium. Therefore, we determined the goblet cell number through Alcian blue/periodic acidic-Schiff staining, which can stain the acidic and neutral mucins of goblet cells. The results confirmed that the goblet cell number was significantly higher in the proximal colon of the *Ndst4^−/−^* mice than in that of their WT littermates (*P* < 0.001; Figure 4A). A colon crypt is composed of several types of cells including stem cells, proliferating cells, differentiating cells, and apoptotic cells. Differentiating cells majorly evolve into secretory and adsorptive cell lineages, which are goblet cells and colonocytes, respectively. To investigate whether an increase in the goblet cell number is accompanied by a decrease in colonocytes, we performed immunohistochemical staining with a colonocyte-specific marker, carbonic anhydrase I. Consistently, the colonocyte number was significantly decreased in the proximal colon of the *Ndst4^−/−^* mice compared with the WT mice (*P* < 0.001; Figure 4B). These results suggested that Ndst4 deficiency disturbs cell lineage differentiation of the colonic epithelium.

### Ndst4 deficiency increases the apoptosis of epithelial cells in the proximal colon

**Figure 5:**
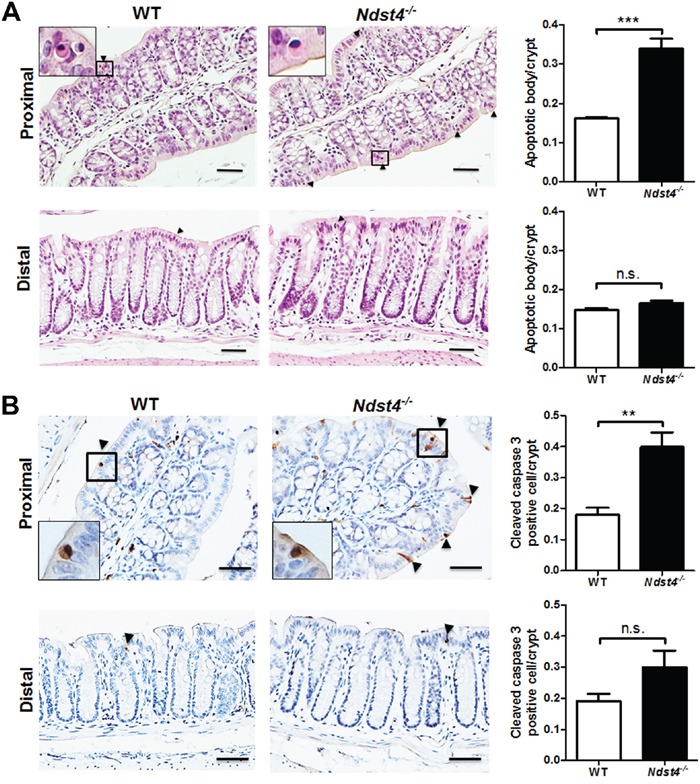
Ndst4 deficiency increases apoptosis along the surface epithelium in the proximal colon Histological analysis was performed on the Swiss rolls made from the proximal and distal colonic segments of the *Ndst4^−/−^* mice and their wild-type (WT) littermates at 10–11 weeks of age (n = 10 for each group). **A.** Hematoxylin and eosin stained sections reveal an increase of apoptotic bodies in the proximal colon of the Ndst4-null mice compared with the WT mice. Arrowheads indicate the apoptotic bodies. **B.** Apoptotic cells in the colonic mucosa were determined using immunohistochemistry for activate caspase-3. In the proximal colon, the apoptotic cells staining brown were significantly increased in the Ndst4-null mice compared with the WT mice. Arrowheads indicate the cleaved caspase-3-positive cells. Insets show higher-magnification views. Fifty crypts were randomly selected in each mouse to count apoptotic bodies and cleaved caspase-3-positive cells. Data represent the mean ± SEM of 10 mice per group. Scale bar, 20 μm. **P < 0.01. ***P < 0.001. n.s., not significant.

Apoptotic bodies, which are regularly pushed to the top of crypts for their removal, are old and nonfunctional cells in the colonic epithelium. In this study, H&E staining of tissue sections revealed that Ndst4 deficiency increased the number of apoptotic bodies in the proximal colon (Figure 5A). To confirm this finding, we performed immunohistochemical staining with an apoptotic marker, cleaved caspase-3. The results showed that the number of cleaved caspase-3-positive cells was significantly increased in the *Ndst4^−/−^* mice compared with the WT mice (*P* < 0.01; Figure 5B), suggesting that Ndst4 is involved in colonic epithelial homeostasis.

## DISCUSSION

The knockout mouse models of *Ndst* genes are useful tools for studying the roles of HSPGs in regulating key developmental signaling pathways. Except for Ndst4, individual knockout mouse models for the other three isoforms have been established [12–16]. In the present study, we first generated an Ndst4-deficient mouse strain and then explored its phenotypic characteristics. Homozygous null mice were viable and fertile and exhibited behaviors and life spans indistinguishable from their WT littermates. Nevertheless, mice lacking Ndst4 exhibited a phenotype restricted to the colonic epithelium. Compared with their WT littermates, the *Ndst4^−/−^* mice exhibited a significantly increased number of goblet cells and a decreased number of colonocytes in the proximal colon. Moreover, Ndst4 deficiency augmented the apoptosis of epithelial cells in the proximal colon. The results suggest that Ndst4 contributes to the heparan sulfate modification of specific HSPGs involved in the differentiation and homeostasis of the colonic epithelium.

In the present study, we confirmed that the expression of *Ndst4* is abundant in the adult mouse brain, which has also been demonstrated by Aikawa *et al.* [11] and Pallerla *et al*. [16]. However, no histological changes were observed in the brain regions (cerebrum, cerebellum and hippocampus) of the Ndst4-null mice in the present study, and no behavioral differences were observed between the *Ndst4^−/−^* mice and their WT littermates. Previous studies have shown that only Ndst1-deficient mice exhibit cerebral and pulmonary hypoplasia, resulting in embryonic and neonatal death [12, 13, 22]. These findings indicate that Ndst1, which is responsible for the development of the brain and lung, exerts a systemic effect on heparan sulfate biosynthesis and exhibits compensatory activities for other Ndst isoforms. In this study, we determined, for the first time, the expression of *Ndst4* in the gastrointestinal tract by using quantitative RT-PCR. Although the relative expression levels of *Ndst1* and *Ndst2* were markedly higher than those of *Ndst4* in the colon (data not shown), altered epithelial differentiation was still observed in the *Ndst4* knockout mice, suggesting that Ndst4 plays a unique role in the colon, and that the loss of its function cannot be compensated for by other Ndst isoforms. A Golgi enzyme complex, the “GAGosome”, which is committed to the assembly of heparan sulfate, is an appealing concept for explaining the molecular machinery responsible for the elongation and modification of glycosaminoglycan chains [23, 24]. Differences in the stoichiometry and composition of the complexes can lead to different modification patterns of the heparan sulfate chains [25]. Thus, the substrate specificity of Ndst enzymes might also be a crucial factor influencing the outcome of biosynthesis. By using mice with targeted mutations in *Ndst1* and *Ndst2*, Ledin *et al.* reported that Ndst1 was preferred as a GAGosome component in WT liver cells, and the presence of Ndst2 in the embryonic and adult liver did not affect the heparan sulfate structure when Ndst1 was also present [26]. However, Ndst2 is the dominating isoform in the heparin synthesis machinery of mast cells; thus, the number of tissue mast cells in mice with Ndst2 deficiency decreases along with dramatically reduced granules in the formed mast cells [14, 27]. GAGOsomes with different combinations of enzymes and their isoforms regulate the synthesis of cell-specific combinatorial structures. On the basis of the study findings, we propose that Ndst4 deficiency might impede the formation of specific GAGosomes and then alter certain HSPG biosynthesis processes required for the differentiation of the colonic epithelium. In addition, the expression levels of *Ndst4* in the ovary, lung, and pancreas were similar to those in the colon. However, Ndst4-null mice were fertile and exhibited normal development of these organs, in which Ndst1 and Ndst2 were highly expressed, and in contrast to the colon, the other Ndst isoforms in these organs could compensate for the lack of Ndst4 activity. Ndst4-deficient mice are a valuable tool that could be used to further generate compound mutants to probe the compensatory Ndst activities and GAGosome function in heparan sulfate biosynthesis *in vivo*.

Colonic mucus secreted by goblet cells serves as a habitat for commensal flora and can prevent bacterial infiltration and subsequent inflammation, thus maintaining barrier integrity [28]. Nevertheless, mucus hypersecretion in mice exhibiting an increase in intestinal secretory cell lineages is accompanied by the alteration of the mucus-associated gut microbiota, which likely causes chronic intestinal inflammation [29]. Moreover, carbonic anhydrases, which are abundant and active in colonocytes, maintain the luminal concentration of bicarbonates required for ion exchange activity [30]. Consequently, decreased expression of colonic carbonic anhydrases may result in diarrhea in mice [31]. In addition, apoptosis of intestinal epithelial cells is a tightly regulated physiological process. Excessive cell apoptosis might reduce intestinal barrier integrity and therefore alter the homeostatic and inflammatory conditions in the colon [32]. In the present study, the Ndst4-null mice exhibited altered cell lineage commitment with an increase in goblet cell number and a decrease in colonocyte number, as well as increased basal-level apoptosis in the colonic epithelium. Accordingly, the results might predict that a colitis-prone phenotype occurs following Ndst4 deficiency, which is being investigated using the genetically engineered mouse strain generated in the present study.

The extracellular matrix is composed of three major proteins (proteoglycans, collagens, and glycoproteins), and the remodeling of the extracellular matrix is responsible for the complex control of differentiation and morphogenesis in various organs [33, 34]. During intestinal development, the distributions of HSPGs at the epithelial–mesenchymal interface display spatial and temporal alterations, indicating their essential roles in this process [35–37]. Heparan sulfate, a linear polysaccharide on the cell surface, facilitates development-associated signaling transduction, such as fibroblast growth factor, Wnt, and sonic hedgehog signalings [38]. Mice with heparan sulfate deficiency showed disrupted Wnt signaling in small intestinal crypts, resulting in the loss of epithelial repair ability after injury [39]. Syndecans and glypicans are two major HSPGs expressed in the colonic epithelium and are associated with cell polarity [40]. Syndecan-1 is one of the most relevant HSPGs in the colon and the most thoroughly characterized HSPG; it binds to various components of the extracellular matrix [41]. In addition, the expression of OCI-5, the rat homolog of human glypican 3, is tightly regulated during intestinal morphogenesis [42]. These findings reveal that HSPGs have pivotal roles in intestinal development. Accordingly, we performed an immunohistochemical study to ascertain whether Ndst4 deficiency alters syndecan-1 biosynthesis in the colon; the results showed no significant difference in syndecan-1 expression in the colonic mucosa between the *Ndst4^−/−^* and WT mice (unpublished data). Future studies should explore the specific HSPGs modified by Ndst4 in the colonic epithelium.

In the present study, we generated, for the first time, an *Ndst4* knockout mouse strain and demonstrated that Ndst4 deficiency could alter the cell lineage commitment in the colonic epithelium, suggesting a crucial role for Ndst4 in the differentiation of colonic progenitor cells. Using the generated mouse strain, we will obtain further insight into the involvement of Ndst4 in intestinal diseases through dextran sulfate sodium-induced colitis and azoxymethane/dextran sulfate sodium-induced tumorigenesis models, which are particularly useful tools for elucidating the tumor suppressor roles of NDST4 in the tumorigenesis and progression of human colorectal cancer.

## MATERIALS AND METHODS

### Ethics statement

All protocols for animal studies were reviewed and approved by the Institutional Animal Care and Use Committee at the National Taiwan University College of Medicine and College of Public Health, Taiwan.

### Targeting vector construction, gene disruption and genotyping of Ndst4-deficient mice

Two targeting vectors were generated to delete exons 2 to 14 of the *Ndst4* gene by the recombineering-based method (Figure S1) [21]. The mouse genomic DNA fragments for insertion of *LoxP* sites in the regions upstream of exon 2 and downstream of exon 14 of *Ndst4* gene were retrieved from the 129/Sv-derived bacterial artificial chromosome clones, bMQ-275F12 and bMQ-68M8, respectively, purchased from the Sanger Institute, UK. The two DNA fragments were inserted separately into a retrieving vector PL253, which contains an *MC1*-driven thymidine kinase cassette for negative selection of ES cells. The resulting constructs were used as the backbones for individual insertion of a neomycin (Neo) resistance cassette flanked by two *LoxP* sites upstream of exon 2 (named Ndst4-1.1), as well as a Neo cassette flanked by two *Frt* sites and one *LoxP* site downstream of exon 14 (named Ndst4-1.2), from PL452 and PL451 vectors, respectively. The PCR primers used for generating the homology arms to retrieve the *Ndst4* genomic DNA (AU, BD, YU, ZD) and insert Neo resistance cassettes (CU, DD, EU, FD) are listed in Supplementary Table S5.

After linearization, the vectors were sequentially used to target 129/Sv-derived ES cells. Neomycin-resistant ES cells were screened for following Cre-mediated homologous recombination. Heterozygous targeted ES clones were identified by Southern blotting. Germ-line chimeras were obtained by injection of the correctly targeted ES cell clones into C57BL/6 blastocysts, and then the founder male chimeras were crossed into a C57BL/6 background. All animals were bred and maintained in a specific pathogen-free environment at the Laboratory Animal Center, National Taiwan University College of Medicine, Taiwan.

The genotypes of the offspring from intercrosses of heterozygous animals were determined by multiplex PCR and further confirmed by Southern blotting of DNA extracted from tail biopsy samples. The multiplex PCR for routine genotyping was performed under the following cycling conditions: a pre-PCR incubation step at 95°C for 10 minutes; 34 cycles of 95°C for 10 seconds, 60°C for 45 seconds, and 72°C for 45 seconds; followed by a final extension of 72°C for 2 minutes (Supplementary Table S6). The amplified products were analyzed by agarose gel electrophoresis. For Southern blot analysis, a probe targeting on the intron 1 of *Ndst4* gene was synthesized by PCR using the PCR DIG Labeling Mix^PLUS^ (Roche Diagnostics, Mannheim, Germany) (Supplementary Table S6). For DNA hybridization, 15 μg of genomic DNA was digested by *Eco*RV, and then separated by agarose gel electrophoresis. After DNA transfer, the nylon membrane was incubated with probes in the DIG Easy Hyb buffer (Roche Life Science) overnight, followed by washing steps. The membrane was incubated with anti-DIG antibody (Roche Life Science) for one hour, and then the hybridized probe was detected with a chemiluminescent substrate CSPD (Roche Life Science).

### RNA extraction

Total RNA was extracted from the frozen tissues of mice, including cerebrum, cerebellum, hippocampus, lung, heart, stomach, duodenum, jejunum, ileum, cecum, colon, liver, pancreas, kidney, ovary, testis, skeletal muscle, thymus, spleen and leukocytes by using TRIzol reagent (Invitrogen, Carlsbad, CA, USA) according to the manufacturer's instructions. The concentration and purity of RNA were determined using NanoDrop ND-1000 spectrophotometer (Thermo Scientific, Hudson, NH, USA), and RNA integrity was confirmed by agarose gel electrophoresis.

### RT-PCR

Cerebral RNA from the *Ndst4^−/−^* and WT mice was used for detection of *Ndst4* gene expression by RT-PCR. Complementary DNA (cDNA) was reverse-transcribed from total RNA (2 μg/20 μL reaction) by using the High Capacity cDNA Reverse Transcription Kit (Applied Biosystems, Foster, CA, USA). Reverse transcription was conducted under the following conditions: 25°C for 10 minutes, 37°C for 2 hours, and 85°C for 5 minutes. The resultant cDNA was diluted 5-fold with diethylpyrocarbonate (DEPC)-treated H_2_O. The primers for detection of *Ndst4* and *β-Actin* mRNA are listed in Supplementary Table S6. PCR amplification was conducted in a final volume of 25 μL by using 2.5 μL of diluted cDNA, 1 μM of each of respective primers, 250 μM of each dNTP, and 1 unit of Super-Therm Gold DNA Polymerase (Bertec Enterprise, Taipei, Taiwan). PCR was performed under the following cycling conditions: a pre-PCR incubation step at 95°C for 10 minutes; 35 (*Ndst4*) or 28 (*β-Actin*) cycles of 95°C for 15 seconds, 55°C for 45 seconds, and 72°C for 45 seconds; followed by a final extension of 72°C for 3 minutes. The amplified fragments were analyzed by agarose gel electrophoresis.

### Quantitative RT-PCR

For the quantification of *Ndst4* RNA expression in mouse organs and tissues, a TaqMan Gene Expression Assay (Mm00480767_m1, Applied Biosystems) was used with a reference gene *Gapdh* (Mm99999915_g1, Applied Biosystems) as a control for RNA quality and quantity. The cDNA was synthesized as mentioned in the RT-PCR section. The reaction mixture included 5 μL of the diluted cDNA, 1 μL of a hydrolysis probe mixture, 10 μL of TaqMan Universal Master Mix (Applied Biosystems), with the addition of DEPC-treated H_2_O to a final volume of 20 μL. Quantitative PCR data were captured by an ABI 7700 Sequence Detection System and analyzed by ABI 7700 SDS Software (Applied Biosystems). The reactions were conducted in duplicate under the following cycling conditions: an incubation step at 50°C for 2 minutes; and an enzyme activation step at 95°C for 10 minutes, followed by 40 cycles of 95°C for 15 seconds and 60°C for 1 minute. The expression levels of *Ndst4* in mouse organs and tissues were normalized to the individual reference gene, *Gapdh*.

### Behavioral and morphological assessment by the modified-SHIRPA protocol

The modified-SHIRPA, consisting of 58 subtests, is a protocol for the comprehensive phenotype assessment of mouse behavior and morphology [43]. For screening of dominant phenotypes in Ndst4-null mice, we implemented the modified-SHIRPA protocol on 27 *Ndst4^−/−^* and 29 WT mice at 8 weeks of age with the help of Taiwan National Laboratory Animal Center. Mice were housed individually per cage and maintained in an incubator with controlled temperature (21 to 22°C) and a reversed light-dark cycle (12 h/12 h) with food and water available ad libitum. All of 58 subtests were executed on a series of six sections in accordance with the scoring system published by Masuya *et al*. [43]. (Supplementary Table S2).

### Hematological assay

For measurement of hematological parameters, blood-EDTA samples were collected from the retrobulbar venous plexus of 26 *Ndst4^−/−^* and 27 WT mice at 8 weeks of age for determination of complete blood counts, including leukocyte differentiation. Hematological parameters were measured by an automatic electronic cell counter, Abbott CELL-DYN 3700 System (Santa Clara, CA, USA) (Supplementary Table S3).

### Serum biochemical assay

For clinical biochemistry tests, whole blood samples were collected from the retrobulbar venous plexus of 26 *Ndst4^−/−^* and 27 WT mice at 9–10 weeks of age. Sera were prepared immediately after blood coagulation and analyzed by a FUJI DRI-CHEM 4000i Analyzer (Fujifilm, Tokyo, Japan) with appropriate reagent kits. The serum biochemical parameters included four metabolites, four enzymes, lipid profile, renal function profile, and two electrolytes (Supplementary Table S4).

### Histological examination

An experienced veterinarian pathologist performed the necropsy of the *Ndst4^−/−^* and WT mice at 10–11 weeks of age (n = 10 for each group). For the preparation of histological sections, mice were fixed by cardiac perfusion with 4% paraformaldehyde in phosphate-buffered saline (PBS), pH 7.4. The colon was removed and divided into proximal and distal parts. Each segment was rolled as “Swiss roll” and processed for histology. The other organs and tissues, including brain, lung, heart, stomach, small intestine, liver, pancreas, kidney, thymus, spleen, skeletal muscle, testis and ovary, were removed, fixed and embedded in paraffin. Serial tissue sections of 5-μm thickness were stained with H&E for histological examination, and processed for other staining.

### Alcian blue and periodic acid-schiff staining

Goblet cell staining was carried out according to the method published by Yamabayahi [44] with some modifications. For Alcian blue staining, tissue sections were deparaffinized in xylene, and then rehydrated through an ethanol gradient to water. Alcian blue 8GX (Sigma-Aldrich, St. Louis, MO, USA) was applied to tissue sections for 15 minutes at room temperature (RT), followed by a 2-minute wash in running tap water. The Alcian blue -stained tissue sections were treated with periodic acid for 5 minutes at RT, washed in distilled water, and then stained with Schiff's reagent (Muto Pure Chemicals, Tokyo, Japan) for 10 minutes at RT, followed by a 5-minute wash in warm water. The tissue sections were counterstained with hematoxylin, and washed under a running tap water for 2 minutes, followed by stepwise dehydration with 95% ethanol twice and 100% ethanol twice for 2 minute each.

### Immunohistochemical staining

Antibody staining was performed on serial 5-μm sections after microwave-mediated antigen retrieval in 10 mM citrate buffer, pH 6.0 by heating for 5 minutes. Peroxidases were quenched with 3% H_2_O_2_ for 10 minutes. Slides were blocked in 5% non-fat milk in PBS for 30 minutes, followed by incubation with anti-carbonic anhydrase I antibody (1:500 dilution; Santa Cruz biotechnology, CA, USA) for 16 hours at 4°C. Detection was performed using the Super Sensitive Polymer-HRP IHC Detection System (BioGenex, San Ramon, CA, USA) according to the manufacturer's instruction. Briefly, sections were incubated with Super Enhancer for 30 minutes and polymer-HRP for 30 minutes, followed by visualization with 3,3’-diaminobenzidine chromogen for 5 minutes. Finally, sections were counterstained with Mayer's hematoxylin (Sigma-Aldrich), dehydrated, and coverslipped.

### Statistical analysis

All data were analyzed for their parametrical distribution by using the Shapiro-Wilk test. The Student's *t*-test was used for between-group analysis, while the Mann-Whitney *U* test was used to compare differences between two independent groups when the dependent variable was distributed nonparametrically. A 2-sided p value less than 0.05 was considered significant for all statistical calculations. Data processing was performed with the SPSS 17.0 (SPSS, Chicago, IL, USA).

## SUPPLEMENTARY MATERIALS FIGURES AND TABLES





## Abbreviations

cDNA: complementary DNA
DEPC: diethylpyrocarbonate
ES cell: embryonic stem cell
H&E: hematoxylin and eosin
HSPG: heparan sulfate proteoglycan
NDST: *N*-deacetylase/*N*-sulfotransferase
*Ndst4^−/−^*: *Ndst4* knockout
Neo: neomycin
PBS: phosphate-buffered saline
RT: room temperature
RT-PCR: reverse transcription-polymerase chain reaction
WT: wild-type.
