# Supplementary material for: Alteration of colonic epithelial cell differentiation in mice deficient for glucosaminyl N-deacetylase/N-sulfotransferase 4
Source: Oncotarget. 2016 Oct 26;7(51):84938–50. doi: 10.18632/oncotarget.12915 (PMC5356710; doi:10.18632/oncotarget.12915)
Supplement: Supplementary file 1 [file oncotarget-07-84938-s001.pdf]

Alteration of colonic epithelial cell differentiation in mice deficient for glucosaminyl *N*-deacetylase/*N*-sulfotransferase 4

SUPPLEMENTARY FIGURE AND TABLES

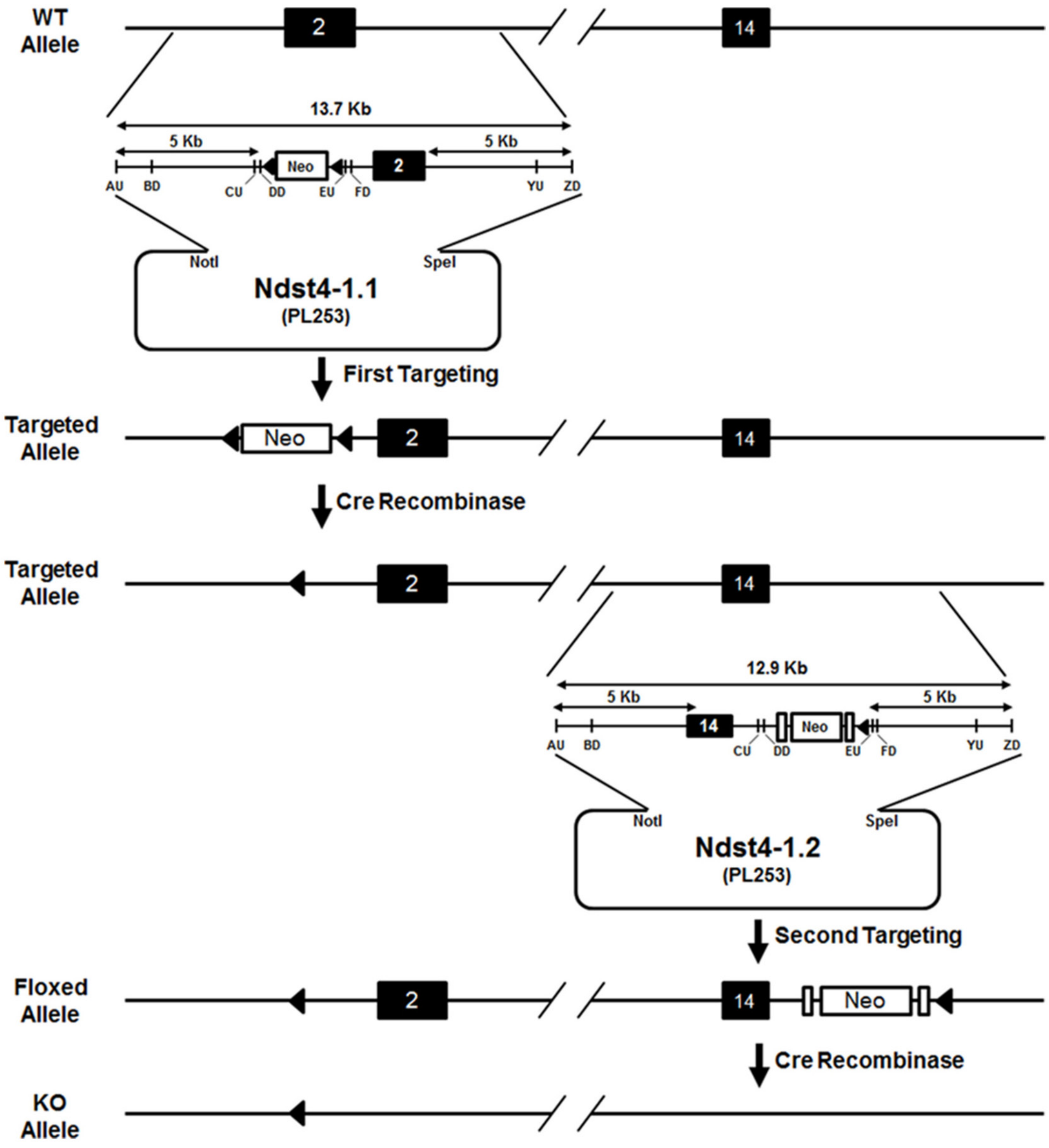

Supplementary Figure S1: Targeting strategy of *Ndst4* knockout mice. Two recombineering targeting vectors were designed to delete the full-length coding region of *Ndst4*. The mouse genomic DNA fragments of *Ndst4* gene were retrieved from the 129/Sv-derived bacterial artificial chromosome clones into PL253 vector via NotI and SpeI sites. The Neo cassette flanked by two *LoxP* sites was introduced upstream of exon 2, as well as the *Frt*-Neo-*Frt*-*LoxP* cassette was inserted downstream of exon 14, sequentially. Homologous recombination was occurred by 5-kb homology arms. Triangle indicates *LoxP* site and open box indicates *Frt* site.

Supplementary Table S1: Normal development and fertility of *Ndst4*$^{+/-}$ and *Ndst4*$^{-/-}$ mice

Parents	Number of productive mating	Total litters (Mean litter size)	Total pups	Sex ratio (Female/Male)	Genotype of *Ndst4*, number (%)		
					+/+	+/–	–/–
Ndst4$^{+/-}$ x *Ndst4*$^{+/-}$	11 male, 22 female	114 (6.0)	683	0.85 (313/370)	175 (25.6)	358 (52.4)	150 (22.0)
Ndst4$^{-/-}$ x *Ndst4*$^{-/-}$	2 male, 4 female	11 (5.5)	60	0.94 (29/31)	0	0	60 (100.0)

Supplementary Table S2: Physiological and behavioral analyses of *Ndst4*$^{-/-}$ and wild-type mice by the modified-SHIRPA protocol

See Supplementary File 1

Supplementary Table S3: Hematology of *Ndst4*$^{-/-}$ and wild-type mice

Parameter	Wild-type (n = 27)	*Ndst4*$^{-/-}$ (n = 26)	*P* value[a]
Leukocytes (x10^3/µL)	7.6 ± 2.4	7.6 ± 1.9	>.99
Neutrophil (/µL)	1297.1 ± 878.9	971.2 ± 604.5	.12
Lymphocyte (/µL)	5934.8 ± 2121.7	6298.8 ± 1985.3	.52
Monocyte (/µL)	110.5 ± 133.5	71.9 ± 120.0	.27
Eosinophil (/µL)	109.6 ± 124.8	102.5 ± 88.7	.81
Basophil (/µL)	121.1 ± 69.4	120.7 ± 65.9	.98
Neutrophil (%)	17.3 ± 11.3	13.6 ± 10.2	.22
Lymphocyte (%)	78.4 ± 11.4	82.2 ± 11.6	.23
Monocyte (%)	1.4 ± 1.7	1.1 ± 1.9	.51
Eosinophil (%)	1.3 ± 1.1	1.3 ± 1.1	.80
Basophil (%)	1.6 ± 0.7	1.7 ± 1.0	.73
Erythrocytes (x10^6/µL)	10.2 ± 0.7	10.2 ± 0.6	.89
Hemoglobin (g/dL)	15.5 ± 0.9	15.5 ± 0.7	.92
Hematocrit (%)	53.6 ± 3.2	53.7 ± 2.6	.84
MCV (fL)	52.5 ± 1.4	52.5 ± 1.2	.95
MCH (pg)	15.2 ± 0.3	15.1 ± 0.4	.54
MCHC (g/dL)	28.9 ± 0.6	28.8 ± 0.7	.50
Platelets (x10^3/µL)	784.6 ± 156.3	756.4 ± 167.4	.53

[a]Student's *t*-test.
Data are the mean ± SD.
MCV, mean corpuscular volume; MCH, mean corpuscular hemoglobin; MCHC, mean corpuscular hemoglobin concentration.

Supplementary Table S4: Serum biochemistry of *Ndst4*$^{-/-}$ and wild-type mice

Parameter	Wild-type (n = 27)	*Ndst4*$^{-/-}$ (n = 26)	*P* value[a]
Metabolites			
Glucose (mg/dL)	176.6 ± 59.5	162.4 ± 34.1	.30
Total protein (g/dL)	5.2 ± 0.4	5.2 ± 0.4	.55
Albumin (g/dL)	3.0 ± 0.4	2.9 ± 0.3	.21
Total bilirubin (mg/dL)	0.9 ± 0.2	0.8 ± 0.1	.24
Enzymes			
Alkaline phosphatase (U/L)	375.9 ± 107.9	389.8 ± 106.1	.64
Aspartate aminotransferase (U/L)	70.1 ± 22.5	66.8 ± 24.3	.62
Alanine aminotransferase (U/L)	36.9 ± 18.6	35.2 ± 10.6	.69
Lactate dehydrogenase (U/L)	468.7 ± 157.6	411.8 ± 193.0	.27
Lipid profile			
Total cholesterol (mg/dL)	82.7 ± 19.9	79.8 ± 18.6	.59
Triglyceride (mg/dL)	114.0 ± 33.0	109.0 ± 19.0	.50
High density lipoprotein (mg/dL)	73.3 ± 14.3	68.7 ± 16.3	.28
Renal function profile			
Blood urea nitrogen (mg/dL)	24.9 ± 3.9	23.3 ± 4.7	.17
Creatinine (mg/dL)	0.2 ± 0.1	0.2 ± 0.1	.75
Uric acid (mg/dL)	2.1 ± 0.6	2.2 ± 0.6	>.99
Electrolytes			
Ca^{2+} (mg/dL)	6.3 ± 3.0	5.7 ± 3.2	.47
Inorganic phosphate (mg/dL)	8.6 ± 1.4	8.6 ± 1.7	>.99

[a]Student's *t*-test.
Data are the mean ± SD.

Supplementary Table S5: Primer sequences for generation of homology arms in targeted disruption of the *Ndst4* gene

Primer	Sequence (5' to 3')
**Ndst4-1.1 (bMQ-275F12)**	
**AU**	ata gcggccgC ATAGGTTTGGAGAGGGAA
**BD**	ata aagctt ata cttaag TCAAGTACAGACCAGCAACA
**CU**	ata gtcgaC TCCCTACCTACAAGCCTG
**DD**	ata gaattc ATGCCTGTGTTCTCAGACA
**EU**	ata ggatcC ATTGTGGTGTATGGATCG
**FD**	ata gcggccgc TCTGACATAAGATGGTGACCT
**YU**	ata aagctt GCATGCACATATATTGGTGT
**ZD**	ata actagt CCACTGATGCAATAGAGACA
**Ndst4-1.2 (bMQ-68M8)**	
**AU**	ata gcggccgc TCTTCTGAGTTGATGTGTTCC
**BD**	ata aagctt ata cttaag CACTTTCTTCTTTGATGGGA
**CU**	ata gtcgac AGGGCTAATGTTTCTAAGCA
**DD**	ata gaattc ATGTCTTACAGAGAAGGCTCA
**EU**	ata ggatcc TGTCCATCTATTTGCAAGC
**FD**	ata gcggccgc ATGTTCCCAAAGTCACCTT
**YU**	ata aagctt AAATGTAACCTAGGAGCACG
**ZD**	ata actagt CAATGCTGTGCTGTGAGAT

Additional sequences in lowercase represent the flanking sequences (ata) and restriction sites.

Supplementary Table S6: Primer sequences of RT-PCR, multiplex PCR for genotyping and probe synthesis for Southern blotting

Primer	Sequence (5' to 3')	Amplicon size (bp)
**RT-PCR**		
**Ndst4-F**	GGTAACAACTTGAACTTTTGGTTG	293
**Ndst4-R**	CTCAGCAGAAGGTCGTCACC	
**Actb-F**	TTTGCAGCTCCTTCGTTG	419
**Actb-R**	TCAAACATGATCTGGGTCATC	
**Multiplex PCR**		
**Ndst4-F**	TCCCTACCTACAAGCCTG	
**Ndst4-R**	TCTGACATAAGATGGTGACCT	514 (WT)
**Ndst4-R'**	ATGTTCCCAAAGTCACCTT	741 (KO)
**Probe of Southern blotting**		
**p-Ndst4-F**	GGCATCAAGGAGGTACTTATG	498
**p-Ndst4-R**	CCTAAAGGCTGCAAATTTATATGC	

F, forward primer; R and R', reverse primer; WT, wild-type allele; KO, knockout allele.
