# Supplementary material for: Alteration of colonic epithelial cell differentiation in mice deficient for glucosaminyl N-deacetylase/N-sulfotransferase 4
Source: Oncotarget. 2016 Oct 26;7(51):84938–50. doi: 10.18632/oncotarget.12915 (PMC5356710; doi:10.18632/oncotarget.12915)
Supplement: Supplementary file 2 [file oncotarget-07-84938-s002.docx]

**Supplementary Table S2. Physiological and behavioral analyses of *Ndst4^-/-^* and wild-type mice by the modified-SHIRPA protocol**

| Test name | Wild-type (n = 29) | *Ndst4^-/-^* (n = 27) | *P* value^a^ |
| --- | --- | --- | --- |
| Body weight (g) | 20.7 ± 3.4 | 20.4 ± 2.8 | .72^b^ |
| In the viewing jar |  |  |  |
| Coat color | 0 (0-3) | 0 (0-0) | >.99 |
| Hair length | 0 (0-0) | 0 (0-0) | >.99 |
| Hair morphology | 0 (0-0) | 0 (0-0) | >.99 |
| Respiration rate | 2 (2-2) | 2 (2-2) | >.99 |
| Tremor | 0 (0-0) | 0 (0-0) | >.99 |
| Body position | 3 (0-3) | 3 (3-3) | >.99 |
| Spontaneous activity | 2 (1-3) | 2 (1-2) | .85 |
| Defecation | 3 (0-9) | 3 (0-5) | .09 |
| Urination | 0 (0-1) | 0 (0-1) | .50 |
| In the arena |  |  |  |
| Time elapsed before  mouse starts to move (seconds) | 3.41 ± 1.78 | 4.04 ± 1.67 | .15^b^ |
| Locomotor activity | 15.59 ± 4.07 | 15.49 ± 6.56 | .59^b^ |
| Transfer arousal | 2 (2-3) | 2 (2-3) | .89 |
| Piloerection | 0 (0-0) | 0 (0-0) | >.99 |
| Startle response | 1 (1-2) | 1 (0-2) | .20 |
| Gait | 0 (0-1) | 0 (0-1) | .10 |
| Pelvic elevation | 3 (2-3) | 3 (0-3) | .20 |
| Tail elevation | 1 (1-1) | 1 (0-1) | .61 |
| Touch escape | 2 (1-3) | 2 (1-3) | .46 |
| On the arena |  |  |  |
| Positional passivity | 0 (0-0) | 0 (0-0) | >.99 |
| Trunk curl | 0 (0-0) | 0 (0-0) | >.99 |
| Limb grasping | 0 (0-1) | 0 (0-0) | >.99 |
| Visual placing | 2 (2-2) | 2 (2-2) | >.99 |
| Grip strength | 1 (1-2) | 1 (1-2) | .34 |
| Body tone | 1 (1-1) | 1 (1-1) | >.99 |
| Head morphology | 0 (0-0) | 0 (0-0) | >.99 |
| Pinna reflex | 1 (1-1) | 1 (1-1) | >.99 |
| Pinna morphology (Right) | 0 (0-0) | 0 (0-0) | >.99 |
| Pinna morphology (Left) | 0 (0-0) | 0 (0-0) | >.99 |
| Corneal reflex | 1 (1-1) | 1 (0-1) | >.99 |
| Toe pinch | 3 (2-3) | 3 (2-3) | .23 |
| **Supplementary Table S2. Physiological and behavioral analysis of *Ndst4^-/-^* and wild-type mice by the modified-SHIRPA protocol (continued)** | | | |
| Parameter | Wild-type (n = 29) | *Ndst4^-/-^* (n = 27) | *P* value^a^ |
| Above the arena |  |  |  |
| Body length (mm) | 93.1 ± 4.7 | 93.2 ± 4.0 | .92^b^ |
| Tail length (mm) | 75.7 ± 5.5 | 75.1 ± 5.2 | .70^b^ |
| Tail morphology | 0 (0-0) | 0 (0-0) | >.99 |
| Lacrimation | 0 (0-0) | 0 (0-1) | >.99 |
| Palpebral closure | 0 (0-0) | 0 (0-0) | >.99 |
| Whisker morphology | 0 (0-0) | 0 (0-0) | >.99 |
| Tooth morphology | 0 (0-0) | 0 (0-0) | >.99 |
| Provoked biting | 1 (1-1) | 1 (1-1) | >.99 |
| Salivation | 1 (1-1) | 1 (1-1) | >.99 |
| Heart rate | 1 (1-1) | 1 (1-1) | >.99 |
| Abdominal tone | 1 (1-1) | 1 (1-1) | >.99 |
| Skin color | 1 (1-1) | 1 (1-1) | >.99 |
| Limb morphology (Fore-Right) | 0 (0-0) | 0 (0-0) | >.99 |
| Limb morphology (Fore-Left) | 0 (0-0) | 0 (0-0) | >.99 |
| Limb morphology (Hind-Right) | 0 (0-0) | 0 (0-0) | >.99 |
| Limb morphology (Hind-Left) | 0 (0-0) | 0 (0-0) | >.99 |
| Limb tone | 1 (0-1) | 1 (0-2) | >.99 |
| In the arena Part 2 |  |  |  |
| Wire maneuver | 0 (0-2) | 0 (0-3) | >.99 |
| Righting reflex | 0 (0-0) | 0 (0-0) | >.99 |
| Contact righting reflex | 1 (1-1) | 1 (1-1) | >.99 |
| Negative geotaxis | 0 (0-3) | 0 (0-3) | >.99 |
| Additional comments |  |  |  |
| Fear | 0 (0-0) | 0 (0-0) | >.99 |
| Irritability | 0 (0-1) | 0 (0-1) | >.99 |
| Aggression | 0 (0-1) | 0 (0-1) | >.99 |
| Vocalization | 1 (0-1) | 1 (1-1) | .67 |
| Bizarre behavior | No | No |  |
| Convulsions | No | No |  |

^a^Mann-Whitney *U* test, unless otherwise noted.

^b^Student’s *t*-test.

Data are the mean ± SD or median followed by range in parentheses.
